# Reactive Chemicals and Electrophilic Stress in Cancer: A Minireview

**DOI:** 10.3390/ht7020012

**Published:** 2018-04-27

**Authors:** Vehary Sakanyan

**Affiliations:** Faculté de Pharmacie, Faculté des Sciences et des Techniques, IICiMed, Université de Nantes, 2 rue de la Houssinière, 44322 Nantes, France; vehary.sakanyan@univ-nantes.fr; Tel.: +33-(0)25-112-5620

**Keywords:** cancer, electrophilic stress, oxidative stress, reactive molecules, high-throughput screening, microarrays, nitro-benzoxadiazole, fluorescence detection

## Abstract

Exogenous reactive chemicals can impair cellular homeostasis and are often associated with the development of cancer. Significant progress has been achieved by studying the macromolecular interactions of chemicals that possess various electron-withdrawing groups and the elucidation of the protective responses of cells to chemical interventions. However, the formation of electrophilic species inside the cell and the relationship between oxydative and electrophilic stress remain largely unclear. Derivatives of nitro-benzoxadiazole (also referred as nitro-benzofurazan) are potent producers of hydrogen peroxide and have been used as a model to study the generation of reactive species in cancer cells. This survey highlights the pivotal role of Cu/Zn superoxide dismutase 1 (SOD1) in the production of reactive oxygen and electrophilic species in cells exposed to cell-permeable chemicals. Lipophilic electrophiles rapidly bind to SOD1 and induce stable and functionally active dimers, which produce excess hydrogen peroxide leading to aberrant cell signalling. Moreover, reactive oxygen species and reactive electrophilic species, simultaneously generated by redox reactions, behave as independent entities that attack a variety of proteins. It is postulated that the binding of the electrophilic moiety to multiple proteins leading to impairing different cellular functions may explain unpredictable side effects in patients undergoing chemotherapy with reactive oxygen species (ROS)-inducing drugs. The identification of proteins susceptible to electrophiles at early steps of oxidative and electrophilic stress is a promising way to offer rational strategies for dealing with stress-related malignant tumors.

## 1. Introduction

The emergence of redox cycling of molecular oxygen in cells serve as safeguarding mechanisms against the toxicity of reactive oxygen species. Numerous studies for nearly five decades have contributed to understanding the role of reactive oxygen species (ROS) in oxidative stress, when the overwhelming production of ROS exceeds the ability of the cellular antioxidant system to neutralize reactive molecules [1,2]. The abundance of intracellular hydrogen peroxide (H_2_O_2_) appears to be the main cause of oxidative stress, which is frequently associated with the development of cancer, neurodegenerative disorders, and autoimmune diseases [3]. In animal cells, metal-dependent superoxide dismutases (SOD) are considered as key enzymes that convert a short lifetime superoxide anion (O_2_^•−^) to the neutrally charged and more stable H_2_O_2_ [4], which moves across the plasma membrane by simple diffusion or aquaporin-facilitated diffusion [5]. H_2_O_2_ can be converted to a short lifetime and more reactive hydroxyl radical (^•^OH), which is responsible for the formation of oxidized DNA base products, such as hydroxy-2′-deoxyguanosine, resulting in mutations and development of cancer [6]. In high doses, ROS react with lipids and proteins leading to tissue damage and cell death, whereas in low doses, nonradical H_2_O_2_ functions as a secondary signal transmitter in cells [7].

Chemical substances synthesized for industry, agriculture, and medicine are potential sources of ROS and, therefore, the exposure of the human organism to pollutants over many years can lead to the development of cancer [8]. Reactive chemicals cause oxidative stress, to which the cell responds by activating different chemo-protective mechanisms. Low molecular weight antioxidants, including multifunctional glutathione (GSH), provide a balance of cellular homeostasis by scavenging an increased amount of intracellular ROS in cancer cells [9]. Moreover, detoxification and antioxidant proteins respond to chemical interventions by modulating the expression mainly at the transcriptional level through the Nrf2/Keap1/ARE pathway [10], and the protein Keap1 senses a wide range of Nrf2-inducing chemicals [11].

The excessive production of ROS is not the only cause associated with pathological processes in cells exposed to chemicals. Exogenous chemicals also generate reactive electrophilic species (referred to as RES) in cells, which react with intracellular nucleophilic substrates including DNA, lipids, and proteins. Investigations of various aspects of electrophilic stress revealed a correlation between the formation of DNA adducts, induction of mutations, and cancer development [12]. In the meantime, studying the early steps of RES generation, their binding to protein targets, and the relationship between oxydative and electrophilic stress are still in the emergence stage. Derivatives of nitro-benzoxadiazole (NBD), potent producers of hydrogen peroxide have been characterized as suicidal molecules in the context of cancer cells [13] and therefore, represent an attractive model to study the intracellular formation of electrophilic molecules. Herein, the recent findings in the formation of reactive species by NBD compounds, which rapidly interact with multiple proteins and impair signal transduction, as early signs of oxidative and electrophilic stress in cancer cells are summarized.

## 2. Epidermal Growth Factor Receptor as a Paradigm for Screening Small Molecule Modulators of Cell Signalling

The extracellular region of membrane-spanning receptor tyrosine kinases (RTKs) is at the forefront of interactions with exogenous chemicals, which possess the potential to affect the signalling cascade in cells [14]. Among the ErbB family of RTKs, an important role belongs to the epidermal growth factor receptor (EGFR), which governs downstream signalling pathways responsible for vital cellular functions such as growth, differentiation, survival, and proliferation. The ligand-promoted dimerization of the extracellular region is necessary for the activation of the ATP-binding site in EGFR and coordinated signalling in physiological conditions [14]. A clue to understanding EGFR auto-phosphorylation was finding that the activation of the ATP-binding site required the action of hydrogen peroxide (H_2_O_2_) produced during a cognate ligand binding to the receptor [15,16,17]. The binding EGF to EGFR generates H_2_O_2_ through the membrane-located NADPH oxidase Nox2; then, H_2_O_2_ reacts with a catalytic cysteine (Cys797) in the ATP-binding site leading to the auto-phosphorylation of tyrosine residues located in the cytoplasmic domain of EGFR [17]. Earlier, H_2_O_2_ was also found to react with a catalytic cysteine in protein tyrosine phosphatase (PTP)-1B and inactivates the enzyme [18]. In both cases, H_2_O_2_ oxidizes cysteine to sulfenic acid in the ATP-binding site. Given that EGFR is a substrate for PTP-1B, the phosphorylation status of the receptor and downstream signalling thereby depends on the activities of two enzymes with opposite functions, phosphorylation and dephosphorylation.

The activation of EGFR has also been demonstrated by the ligand-independent mechanism in cells exposed to exogenous H_2_O_2_ or small particulate matter, diesel exhaust particles, or other chemicals [19,20,21]. Noteworthy, chemical substances in cigarettes also cause an aberrant signal transduction through activation of EGFR contributing to the progression of lung cancer in smokers and even in non-smokers [22].

EGFR dimerization is important for the phosphorylation of the receptor, and the dimerization interface of the extracellular region is of huge interest in searching for chemical compounds affecting cell signalling.

Small molecule microarrays provide high-throughput screening (HTS) of chemical compounds immobilized on a functionalized surface of glass slides [23]. We developed non-covalent immobilization of compounds with different structures on the same slide to avoid biased chemical coupling to a functionalized surface, thus, making putative binding sites in protein targets accessible for all moieties of the spotted compounds [24]. As proof of the concept, this method was demonstrated by screening porphyrin derivatives to select appropriate candidates for photodynamic therapy of tumors [25].

**Figure 1:**
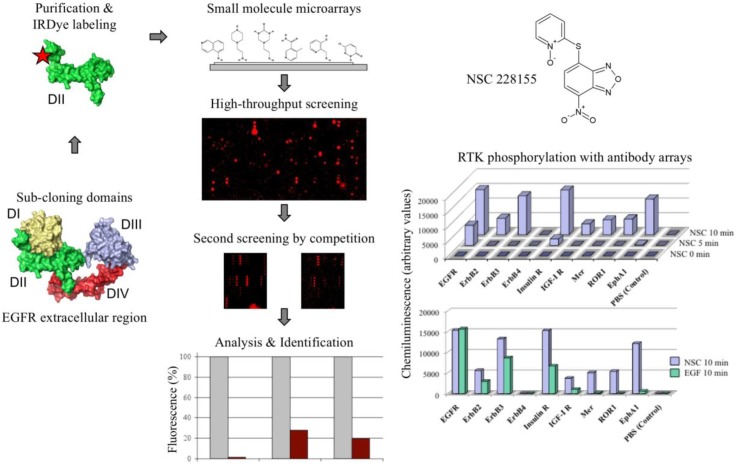
Screening strategy of compound libraries with small molecule microarrays. The extracellular region of epidermal growth factor receptor is shown in the tethered conformation of four domains. The histogram shows competition of selected compounds (for NSC 228155 column 1) for binding to non-labelled and IRDye 800 labelled DII domain of epidermal growth factor receptor (EGFR; brown column). Profiling of membrane-spanning receptor tyrosine kinases (RTK) phosphorylation with compound NSC 228155 was performed with antibody microarrays. Modified with permission from Reference [26].

For screening chemical compound libraries using EGFR as a probe, we took advantage of the domain organization of the EGFR extracellular region by constructing shorter proteins to expose the whole protein surface to interactions with small molecules ([26]; Figure 1). Detection of illuminated spots with near-infrared fluorescence reduced the interference from auto-fluorescent signals emitted by heterocyclic rings of chemicals at visible wavelengths. To explore the ability of the selected compounds to act as potential modulators of EGFR activity, their action was further tested with antibody microarrays and Western blotting.

Screening the Diversity Set II library of the National Cancer Institute allowed us to identify, among 20 pre-selected binders, compound NSC 228155, which remarkably enhanced EGFR tyrosine phosphorylation [26]. The compound contains the scaffold 4-nitro-2,1,3-benzoxadiazole (NBD), which was found to be responsible for the biological consequences in cancer cells. Similar molecules bearing this scaffold were next detected in the French National Chemical Library by virtual screening. A two-ring structure of NBD emits intrinsic fluorescence at visible wavelengths, and this property was employed in the development of molecular probes and fluorescent dyes for cell imaging [27]. However, the biologically relevant consequences of this important class of chemicals were still obscure at the beginning of our investigations.

Analysis of two cell-permeable derivatives, NSC 228155 and CN 009543V, revealed that both compounds stimulated the dimerization of EGFR associated with enhanced tyrosine phosphorylation at the positions Tyr1068 and Tyr1173, and the activation of downstream MAPK/ERK and ubiquitin/proteasome signalling pathways in breast cancer cells [26]. Moreover, EGFR kinase inhibitors prevented tyrosine phosphorylation of the receptor. These results appeared to indicate that the NBD compound binding to the extracellular region promoted EGFR dimerization and activation of the active site as described for the cognate ligand. However, a weak inhibition of EGFR phosphorylation with a neutralizing antibody and the activation of a larger set of RTKs when compared to a cognate ligand EGF (see Figure 1) were in favour of the generation of H_2_O_2_ with NBD compounds differently from the binding EGF, without implication of the extracellular region dimerization of the receptor. In any case, these data demonstrate the vulnerability of EGFR in the perception of external chemical signals leading to aberrant activation of downstream pathways.

## 3. Superoxide Dismutase 1 Plays a Pivotal Role in Cell Signalling Induced with Nitro-Benzoxadiazole Compounds

Generation of intracellular ROS by NBD compounds may be related to the action of superoxide dismutase, catalase, and/or glutathione peroxidase, which are responsible for the protection of human cells against toxic oxygen species [28]. Given that the activity of these enzymes is exhibited by dimeric or higher oligomeric forms, we studied the oligomerization state of corresponding proteins in breast cancer and prostate cancer cells exposed to NBD compounds [29,30]. No changes were detected in the oligomerization or expression of catalase and glutathione peroxidase. On the contrary, a significant proportion of Cu/Zn SOD1, which is mainly located in the cytoplasm and much less in mitochondria [31], was detected as a 32-kDa dimer in cells exposed to lipophilic NBD compounds for 5–10 min. The induced dimers of SOD1 were stable and they did not disrupt during electrophoretic migration in a denaturing gel in contrast to less stable dimers of native enzyme in non-treated cells [29].

Pre-incubation of cells with antioxidants *N*-acetyl-l-cysteine or thioglycerol prevented the induction of SOD1 dimerization and tyrosine phosphorylation of EGFR by lipophilic NBD compounds. The specificity of this relationship was confirmed by the pre-incubation of cells with an irreversible tyrosine kinase inhibitor CI-1033 (binds to Cys797 in EGFR) that abolishes EGFR phosphorylation without the suppression of SOD1 dimerization [29]. Moreover, the inhibitor of Nox2, apocyinin, reduced the phosphorylation of EGFR when the cells were stimulated with EGF, according to the published data [10], but had no effect when cells were treated with NBD compounds.

The stability of the NBD-induced dimers of SOD1 was used to assess the kinetics of EGFR phosphorylation in relation to SOD1 dimerization in the RNA interference experiments. Since the human SOD1 is a long-lived protein [32], a longer cultivation of cells transfected with SOD1 siRNA was necessary to supply in order to strengthen the silencing effect. No EGFR phosphorylation was detected in SOD1 knockdown cells treated with NBD compounds [29]. Meanwhile, the enhanced tyrosine phosphorylation of EGFR correlated in time with an augmentation of the proportion of SOD1 dimers within 15-min exposure of cells transfected with scrambled siRNA.

The importance of the intracellular location of NBD electrophiles in the activation of EGFR was confirmed by in cellulo studies with fluorescent microscopy; only lipophilic derivatives were found to enter cells. Moreover, a real-time monitoring of fluorescence revealed that pre-incubation of cells with catalase suppressed the fluorescence, thereby identifying H_2_O_2_ as the intracellular ROS that accumulated rapidly, within 5 min in breast cancer cells, and only when cells were exposed to lipophilic, but not to non-lipophilic NBD compounds [29].

These data demonstrated that new mechanism of the activation of EGFR relies on coupling the generation of hydrogen peroxide to the stable dimerization of SOD1 with NBD compounds in cytoplasm. Hence, unlike the generation of H_2_O_2_ by Nox2 operating in the ligand-dependent mechanism to activate EGFR, the bound NBD compound/SOD1 dimer fulfils the role of H_2_O_2_ provider in the ligand-independent mechanism. Once the concentration of hydrogen peroxide, catalyzed by SOD1, reaches a critical level, it sulphenylates the cysteine in the catalytic site of EGFR and PTP-1B, and together, the activated protein kinase and inactivated protein phosphatase contribute to enhancing EGFR phosphorylation leading to aberrant signalling in downstream pathways.

The role of SOD1 in cell signaling was also proven for the DNA protein kinase catalytic subunit (DNA-PK) in prostate cancer cells exposed shortly to the compound NSC 228155 [30]. The DNA-PK is a key protein involved in the repair of DNA double-strand breaks, which are considered the most cytotoxic DNA lesions and result from endogenous events such as the production of ROS during cellular metabolism [33]. It was found that tyrosine phosphorylation as well as the expression and activity of DNA-PK drastically decreased in cells exposed to NBD compounds for 10 min. Of note is that this decrease was accompanied by increased protein ubiquitination and the activation of the proteasome machinery leading to the protein degradation [29].

Thus, in the absence of appropriate downstream reactions, catalyzed by catalase and/or glutathione peroxidase, the induced by lipophilic NBD compounds dimers of SOD1 produced an excessive amount of H_2_O_2_, which rapidly impair the signaling functions of protein kinases.

## 4. Lipophilic Electrophiles Rapidly Bind to Multiple Proteins in Cancer Cells

**Figure 2:**
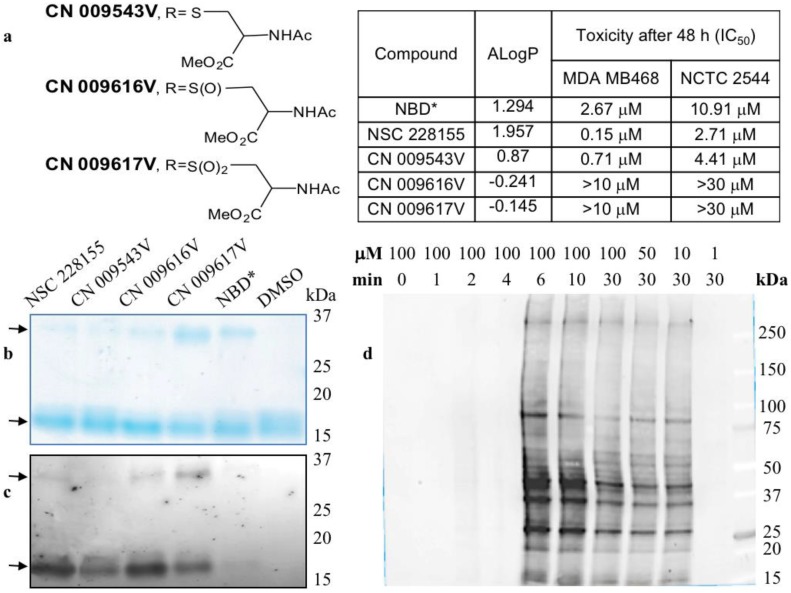
Nitrobenzoxadiazole (NBD) compounds rapidly bind to multiple proteins in cells. (**a**) Structure, lipophilicity, and cytotoxicity of NBD compounds; (**b**) Coomassie staining of the purified Cu/Zn superoxide dismutase 1 (SOD1) bound to NBD compounds; NBD* contains only the NBD scaffold; (**c**) Detection of intrinsic fluorescence emitted by compound-bound monomeric and dimeric forms of a purified SOD1; arrows show monomers and dimers of SOD1; (**d**) Detection of NSC 228155 compound binding to multiple proteins in living breast cancer cells as in (**b**). Fluorescent detection of compound binding to proteins in vitro (**c**) and in vivo (**d**) is described in References [29] and [36], respectively.

Zinc binding promotes dimerization and stabilizes the native form of SOD1, and therefore, the dimerization state of wild type and dimerization-impaired mutants determines the stability and catalytic activity of SOD1 in cells [34]. Both lipophilic and non-lipophilic NBD compounds (Figure 2a) promote the formation of stable dimers of a purified SOD1, and the yield of dimeric forms was higher with non-lipophilic compounds CN 009616V and CN 009617V (Figure 2b). The detection of intrinsic fluorescence emitted by NBD compounds was corroborated with staining data (Figure 2c). This also indicated that both the lipophilic and non-lipophilic compounds were bound to the monomers and cross-linked them into fluorescent dimers of SOD1, strongly suggesting covalent interaction between monomers. Hence, reducing lipophilic CN 009543V or non-lipophilic CN 009616V and CN 009617V, which differ only in the sulfur oxidation state (see Figure 2a), is possible regardless of their lipophilicity outside of cells if conditions are appropriate such as in a slightly alkaline medium to promote the formation of reactive electrophilic species and covalent binding to protein targets. In contrast to NBD-thioether CN 009543V, NBD-sulfoxide CN 009616V and NBD-sulfone CN 009617V can stabilize the electrophiles formed, thus explaining their higher reactivity towards nucleophilic amino acids involved in the formation of the purified SOD1 dimers [29].

The electrophilic status of chemicals a priori suggests a putative electrophile–nucleophile interaction with protein targets. However, the question is how quickly these interactions take place and how to be sure about the authentic nature of such interactions, without using chemical trucks for detection that could distort results. In this regard, the binding of fluorescent chemicals to proteins can be directly visualized in gel by electrophoretic migration and scanning at appropriate wavelengths (in-gel detection) [35]. However, a water environment affects photo-physical properties such as emission spectra, extinction coefficients, and quantum yields, and frequently abolishes the fluorescent signal emitted in gel, and that was a case with NBD compounds. To override the bottlenecks of in-gel detection, we turned to the blotting technique by transferring the total proteins extracted from treated cells onto the nitrocellulose membrane and the further detection of a fluorescent signal at λ_ex_ = 480 nm and λ_em_ = 520 nm using an imaging system equipped with appropriate filters [36].

A large number of proteins were detected as illuminated bands in cancer cells after a short exposure to lipophilic NBD compounds. Nearly 20 proteins, emitting strong and moderate signal and ranging from 15 kDa to about 400 kDa, were detected in cells exposed to NSC 228155 for 6 min (Figure 2d). Of note, the signal intensity and profile of bound proteins were similar, but not identic in breast cancer and lung cancer cell lines suggesting variations in the expression levels of the same proteins and/or differences in proteins to be targeted. Treatment of cells with NBD compounds for a longer period of 30 min and more resulted in the decrease of the fluorescence emitted from almost all proteins reacted at a shorter time. This argued with the activation of the EGFR-mediated ubiquitin-degradation pathway shortly after the exposure of cancer cells to NBD compounds [26]. Straightforward detection of fluorescent molecules illuminating interactions with proteins partners confirmed that only lipophilic NSC 228155 and CN 009543V were bound to proteins in living cells, even after exposure for 1 h [36]. Thus, in spite of the electrophilic potency of NBD derivatives, only structures enabled to overcome the membrane bilayer barrier exhibited the electrophilic power manifested by rapid binding to multiple proteins in cells.

It has been shown that NBD derivatives carrying different lateral chains bind to glutathione *S*-transferase (GST), Myc oncoprotein, and a number of mitochondrial proteins [37,38,39]. Therefore, the data accumulated to date make it possible to state that NBD electrophiles covalently interact with various proteins in cells.

## 5. Electrophilic and Oxidative Stress Are Mutually and Tightly Related Processes

Exogenous chemicals possess various electron-withdrawing groups with different potencies to react with nucleophilic amino acids by accepting a pair of nucleophilic electrons that result in the formation of a covalent bond with proteins [40,41,42]. Different approaches have been developed to determine the products of in vitro chemical reactions using model reactive electrophiles and proteins [43,44]. Remarkable progress has been achieved by studying interactions between a variety of electrophilic chemicals and nucleophilic amino acids in proteins, and by dissecting a cascade of events providing the protection of cells against electrophilic attacks of exogenous chemicals [45,46]. However, the early steps of converting exogenous chemicals to reactive electrophilic species inside the cells are still obscure.

**Figure 3:**
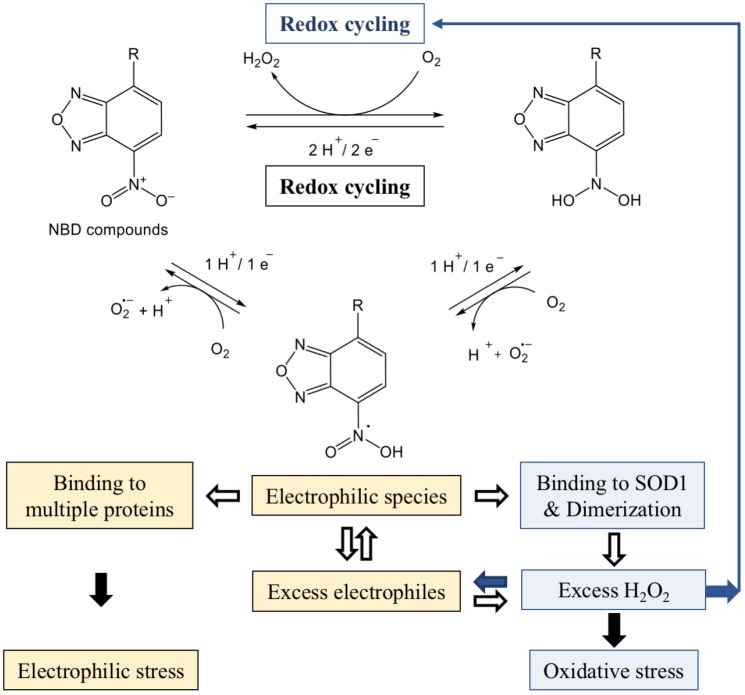
Generation of reactive species by reduction of NBD compounds. Lipophilic compounds generate reactive oxygen species (ROS) and concomitant reactive electrophilic species (RES), which independently react with a variety of proteins. The Cu/Zn superoxide dismutase 1 (SOD1) dimers trigger new rounds of O_2_ redox cycling leading to the production of excess H_2_O_2_ in cancer cells. The reduction of the nitro group is described in Reference [48].

Derivatives of 7-nitro-2,1,3-benzoxadiazole have been described as suicide inhibitors of GSTs [13]; the enzymes involved in the maintenance of homeostasis and considered as attractive targets in anti-cancer therapy [47]. Structural analysis of the protein bound NBD-hexanol derivative revealed its covalent interaction with glutathione in the glutathione *S*-transferase P1, an isoenzyme of the GST superfamily [37]. Thereafter, compound 7-nitro-4(phenylthio)benzofurazan (NBF-SPh) has been found to be a potent generator of superoxide and hydrogen peroxide when compared to other ROS producing chemicals [48]. NBF-SPh promotes the consecutive production of O_2_^−^ and H_2_O_2_ through the reversible electrochemical redox cycling of molecular oxygen in cells (Figure 3). The reduction of the nitro group is associated with the increase in the consumption of O_2_, especially while cells grow at high concentrations of oxygen. Chemical and biophysical analyses have revealed the formation of intermediate products generated during the reduction of the nitro group, most likely reactive electrophiles with scavenging activity. Chemical transformations have indicated that irreversible reduction of NBF-SPh might generate other intermediate electrophilic structures, but with redox potentials too electronegative and incompatible with a biological process. Therefore, reduction of the nitro group should halt through the redox cycling formation of NBF-SPh electrophiles that can conjugate with macromolecular structures in cells [48].

Our results support that NBD compounds are potent generators of ROS and provide missing but important elements for understanding the power of ROS and RES generated simultaneously and leading to aberrant effects in cancer cells (see Figure 3). As described above (Section 3 and Section 4), in the absence of adequate downstream catalytic reactions, SOD1 dimers resulting from covalent binding with NBD compounds rapidly produce excessive amounts of hydrogen peroxide. The explanation for this is the fact that stable and active SOD1 dimers can trigger new rounds of O_2_ redox cycling independent of the nitro group reduction initiated by NBD compounds, thereby producing more H_2_O_2_. In addition, lipophilic NBD compounds simultaneously generate concomitant NBD electrophiles, which rapidly bind to many proteins in cells. Therefore, SOD1 dimer-mediated rounds of redox cycling facilitate the production of excess electrophiles during their depletion due to covalent binding to proteins. It is noteworthy that in comparison to the rapid consumption of high amounts of molecular oxygen, a longer time is required to deplete greater concentrations of electrophiles in cancer cells [48].

Thus, two mutually related entities of redox reactions, ROS and RES, attack a variety of nucleophilic protein targets in cells. The NBD compound binding is dose-dependent, and their intracellular concentration is limited by the transfer through the membrane [29]. If intermediate electrophilic structures do not interact with the targets, unclaimed electrophiles return to the new redox cycle, which is consistent with the scavenging ability of lipophilic compounds [29,48]. However, redox cycling can operate without the participation of NBD compounds beyond the saturation concentration, provided there is a high concentration of oxygen in the cultivation medium. In any case, the possibility of the continuous operation of the oxidation-reduction cycle is the reason for the production of a large amount of ROS by NBD compounds as long as the cells are viable (see Figure 3).

Nitrobenzoxadiazole-promoted toxic doses of H_2_O_2_ have been shown to result in peroxidation of membrane lipids [48]. This corroborates with rapid, within 12 min induction of blebbed, morphologically visible structures [29] associated with detachment of the membrane and cell death [49]. The blebbing was also observed with high concentrations of a physiologically relevant molecule EGF to stimulate EGFR in cancer cells [29]. Since pre-incubation with catalase reduces the membrane detachment induced with NBD compounds or EGF, it can be assumed that reactive O_2_^•−^ and/or H_2_O_2_ rather than intermediate electrophiles are responsible for the detrimental effect of the membrane.

**Figure 4:**
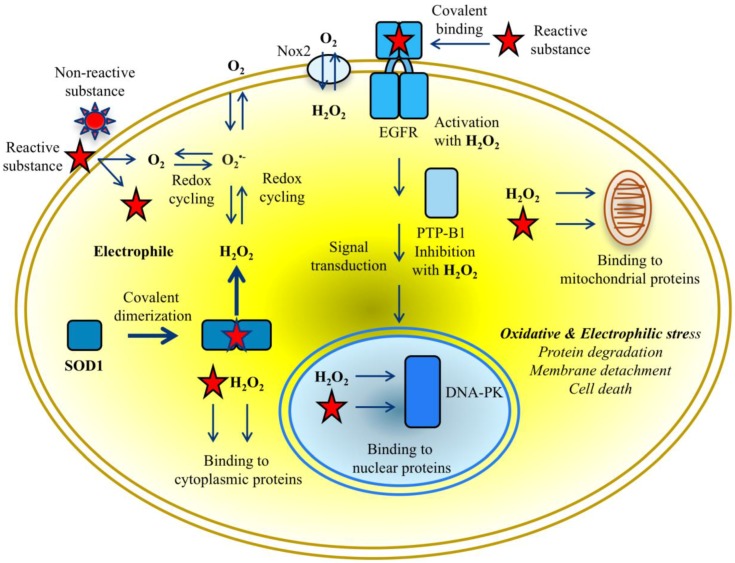
Crosstalk between SOD1 and proteins in cytoplasm, nucleus, and mitochondria mediated by reactive species in cancer cells exposed to putative exogenous chemicals. Overwhelming production of ROS and RES simultaneously causes oxidative and electrophilic stress. Reactive species beyond critical concentrations in cytoplasm lead rapidly to protein degradation and membrane detachment, and ultimately to death of cancer cells.

The chemistry of the interaction of electrophilic NBD compounds with different proteins is not yet clear; it may be caused by the opening of the benzofurazan ring [48], but the participation of the nitro group or sulfur with lateral branches in the chemical reaction with nucleophilic amino acids cannot be ruled out. As far as ROS and RES can compete for reacting with the same amino acids, various factors can affect the priority of the reaction with H_2_O_2_ or electrophiles with protein targets. The dual action of reactive oxygen and electrophilic species aggravates deleterious effects depending on the character of impaired protein functions and the response of cell-protective pathways to reactive molecule species (Figure 4). In particular, NBD compounds affect functions of protein kinases differently; they aberrantly activate EGFR-triggered signalling pathways and inhibit the DNA-PK-mediated DNA repair pathway. The majority of targeted proteins undergo further degradation most likely through ubiquitination/proteasome machinery leading to the apoptotic death of cancer cells [13,29,30,36]. Notably, according to half maximal inhibitory concentration (IC_50_) values lipophilic NBD compounds proved to be more toxic in breast cancer MDA MB468 cells than in transformed NCTC 2544 keratinocytes (see Figure 2a). In contrast, non-lipophilic compounds were clearly non-toxic, due to their inability to penetrate cells and inactivate vital functions [29].

According to our data, the gravity of the electrophilic stress primarily depends on the aberrant state of SOD1 dimers in providing toxic concentrations of ROS during the consumption of oxygen in cells cultivated with NBD compounds. This means that the development of electrophilic stress is dependent on and tightly related to oxidative stress. The progression of two inseparable processes apparently reflects the transition of cells from normastasis to hypostasis that can be measured by the increase of hypoxia in the tumor environment due to a decrease in oxygen level [50]. Given that RES interactions are relatively slower and can be masked by ROS interactions, it makes it difficult to reliably distinguish the proportion of electrophilic stress and “pure oxidative stress” by measuring the rate of hypoxia in the treated cells. Therefore, electrophilic stress appears to be preferably defined as the overwhelming production of reactive electrophilic and oxygen species that exceeds the ability of anti-chemoprotective and anti-oxidant systems to neutralize both types of reactive species. The apparition of stable SOD1 dimers in cells, detectable by Western blot, may be used as an early sign of electrophilic stress in response to the chemical intervention of potent producers of hydrogen peroxide (see Figure 4).

The benzoxadiazole (benzofurazan) scaffold is a popular template for the creation of imaging probes in living cells [27,39] and the development of anticancer drugs [38,47]. It has been suggested that the anti-cancer effects of NBD compounds are related to the action of hydrogen peroxide generated during the redox cycling in cancer cells [48]. This review supports this assumption; moreover, it emphasizes that NBD compounds produce reactive electrophilic species that interact with many proteins in cells. Such promiscuity clearly reduces the specificity of the action on the expected target in the diseased cells [51]. This should be borne in mind when developing target-specific tools and drugs using NBD and similar structures with the NO_2_ group for diagnosis and therapy.

Cancer cells produce higher levels of ROS compared to healthy cells [52], and this difference has been used in the development of ROS-inducing cancer chemotherapy [9,53]. Some drugs, such as quinone derivatives, undergo oxidation and produce concomitant ROS, which cause oxidative stress, and then death of cancer cells [54]. The elucidation of the NBD compound-promoted generation of mutually related reactive species, ROS and RES that behave as functionally independent entities suggests that electrophilic stress also remarkably contributes to the destruction of cancer cells by oxidized ROS-inducing drugs. Moreover, the binding of the electrophilic moiety to multiple proteins resulting in impairing many cellular functions in different types of cancer can explain various and unpredictable side effects in patients undergoing chemotherapy with ROS-inducing drugs. Therefore, the identification of proteins susceptible to reactive electrophilic species is of paramount importance in better understanding the role of electrophilic stress in cancer progression and developing diagnostic and therapeutic biomarkers with the hope of offering more rational strategies for combating malignant tumors.

## 6. Perspectives

The precise definition of oxidative and electrophilic stress requires the knowledge of nucleophilic sites susceptible to both types of reactive species in the human proteome. A cysteine residue is highly sensitive to H_2_O_2_ and its reactivity varies in different protein contexts. Oxidative modification of nucleophilic cysteine into reversible sulfenic acid is of great importance in cellular metabolism [55], and the detection of sulfenylated residues in proteins has been used for the evaluation of global thiol-redox effects of the proteome with mass spectrometry (MS) methods [56,57]. The dissection of the human redoxome has revealed that among the 900 sulfenylated sites, the majority of cysteine residues are in logical relation with antioxidant and metabolic functions in cells [58]. However, a large number susceptible to H_2_O_2_ sites varied from one cell line to another and were less conserved than their redox-insensitive counterparts.

Proteomic identification of targets sensitive to electrophilic chemicals by adductomic methods is a challenge and requires the solution of a number of technical problems. Proteins targeted by fluorescent electrophiles can be, in principle, identified at low resolution following the detection of intrinsic fluorescence emitted by the bound chemicals in cancer cells [36]. Other promising methods described for specific proteins [43,44] need to be scaled and approved to a larger number of potential targets. Recently, Rappaport’s group has demonstrated that sulfenylated cysteine residues can be distinguished from electrophilic adducts in a biological fluid, the serum of workers exposed to benzene [59]. The authors have characterized various modifications induced in human serum albumin and identified benzene-specific Cys34 adducts of reactive oxygen and carbonyl species, probably associated with the development of leukemia in patients. These encouraging data indicate in favour of the possibility to detect proteins differentially targeted by ROS and RES in cancer proteome.

Chemical compounds in pollutants, when the content of reactive molecules is hundreds of times higher than the permissible doses [60], can rapidly penetrate into cells and adversely affect the human organism. In this regard, multifaceted microarrays primarily developed for drug discovery purposes (see Figure 1) are promising tools for the cost-effective assessment of the biological reactivity of a huge number of synthetic molecules, in particular, the ability to bind to detoxification, antioxidant, and signaling proteins and modulate the protein expression and post-translational modifications. Volatile lipophilic or non-lipophilic electrophiles in atmospheric emissions may provoke the depletion of the ozone layer with subsequent undesirable effects on the biological systems on our planet. Undoubtedly, studying electrophiles is an integral part of the global exposome problem [61] in our efforts to prevent the progression of cancer, neurodegenerative, and other severe diseases.
